# Prevention of Triglyceridemia by (Non-)Anticoagulant Heparin(oids) Does Not Preclude Transplant Vasculopathy and Glomerulosclerosis

**DOI:** 10.3389/fcell.2022.798088

**Published:** 2022-03-07

**Authors:** Pragyi Shrestha, Kirankumar Katta, Ditmer Talsma, Annamaria Naggi, Jan-Luuk Hillebrands, Bart van de Sluis, Jacob van den Born

**Affiliations:** ^1^ Department of Nephrology, University of Groningen, University Medical Center Groningen, Groningen, Netherlands; ^2^ Center for Eye Research, Department of Ophthalmology, Oslo University Hospital Ullevål, Oslo, Norway; ^3^ Department of Medical Microbiology, University of Groningen, University Medical Center Groningen, Groningen, Netherlands; ^4^ Ronzoni Institute, Milano, Italy; ^5^ Medical Biology—Pathology Division, University of Groningen, University Medical Center Groningen, Groningen, Netherlands; ^6^ Department Pediatrics, Section Molecular Genetics, University of Groningen, University Medical Center Groningen, Groningen, Netherlands

**Keywords:** chronic transplant dysfunction, heparin, pcsk9, syndecan-1, dyslipidemia

## Abstract

**Background:** In renal transplantation, chronic transplant dysfunction (CTD) is associated with increased PCSK9 and dyslipidemia. PCSK9 is an enzyme that increases plasma cholesterol levels by downregulating LDLR expression. We recently showed increased PCSK9–syndecan-1 interaction in conditions of proteinuria and renal function loss. Treatment with heparin(oids) might be a therapeutic option to improve dyslipidemia and CTD. We investigated the effects of (non-)anticoagulant heparin(oids) on serum lipids, syndecan-1 and PCSK9 levels, and CTD development.

**Methods:** Kidney allotransplantation was performed from female Dark *Agouti* to male Wistar Furth recipients. Transplanted rats received daily subcutaneous injections of saline, unfractionated heparin, and RO-heparin or NAc-heparin (2 mg heparin(oid)/kg BW) until sacrifice after 9 weeks of treatment.

**Results:** Saline-treated recipients developed hypertension, proteinuria, and loss of creatinine clearance (all *p* < 0.05 compared to baseline), along with glomerulosclerosis and arterial neo-intima formation. Saline-treated recipients showed significant increase in plasma triglycerides (*p* < 0.05), borderline increase in non-HDLc/HDLc (*p* = 0.051), and ∼10-fold increase in serum syndecan-1 (*p* < 0.05), without significant increase in serum PCSK9 at 8 weeks compared to baseline. Heparin and non-anticoagulant RO-heparin administration in transplanted rats completely prevented an increase in triglycerides compared to saline-treated recipients at 8 weeks (both *p* < 0.05). Heparin(oids) treatment did not influence serum total cholesterol (TC), plasma syndecan-1 and PCSK9 levels, creatinine clearance, proteinuria, glomerulosclerosis, and arterial neo-intima formation, 8 weeks after transplantation. Combining all groups, increased syndecan-1 shedding was associated with TC (*r* = 0.5; *p* = 0.03) and glomerulosclerosis (*r* = 0.53; *p* = 0.021), whereas the non-HDLc/HDLc ratio was associated with the neo-intimal score in the transplanted kidneys (*r* = 0.65; *p* < 0.001).

**Conclusion:** Prevention of triglyceridemia by (non-)anticoagulant heparin(oids) neither influenced PCSK9/syndecan-1 nor precluded CTD, which however did associate with the shedding of lipoprotein clearance receptor syndecan-1 and the unfavorable cholesterol profile.

## Introduction

Chronic transplant dysfunction (CTD) is a functional decline of the transplanted kidney characterized by a progressive increase in plasma creatinine levels, proteinuria, and hypertension (Van Timmeren et al., 2007). About 30–50% of renal allografts are lost after 5 years of transplantation due to CTD, making CTD a major challenge in the field of transplantation (Marcén and Teruel, 2008; Ekberg and Johansson, 2012; Lai et al., 2021). Histological changes, such as interstitial fibrosis, tubular atrophy, and vascular and glomerular occlusions, are hallmarks of a dysfunctional allograft (Nankivell et al., 2003; Van Timmeren et al., 2007). Among these histopathological changes, transplant arteriopathy and glomerulosclerosis are common and characterized by myo-intimal proliferation, resulting in the formation of an occlusive neo-intima in the intragraft arteries and arterioles, mesangial proliferation, and matrix production, leading to glomerulosclerosis (Shoji et al., 2020; Uffing et al., 2020).

The pathogenesis of CTD is complex and multifactorial, and involves immune and non-immune factors. Human leukocyte antigen (HLA) mismatches between donor and recipient give rise to cytotoxic T cells, NK cells, and donor-specific antibodies, causing immune-related injury to the graft, whereas hypertension, ischemia, infections, dyslipidemia, diabetes, and drug toxicity cause non-immune-related graft injury (Nankivell et al., 2003; Najafian and Kasiske, 2008). Among these non-immune-related factors, dyslipidemia is an important but often overlooked factor in the development and progression of CTD and cardiovascular diseases (Del Castillo et al., 2004; Luca et al., 2015; Elkins et al., 2019). Moreover, dyslipidemia can be modified by pharmacological intervention, and to some extent, by lifestyle modification, making dyslipidemia the potentially effective target to prevent CTD. It has been shown that pravastatin, an HMG-CoA reductase inhibitor, lowered total cholesterol levels and reduced the development of coronary vasculopathy in cardiac transplant patients (Mahle et al., 2005). Studies on animals with lupus nephritis, aminonucleoside nephrosis, reduced renal mass, diabetes mellitus, or systemic hypertension have shown that cholesterol can increase the incidence of glomerulosclerosis (Agrawal et al., 2018).

Dyslipidemia is characterized by increased serum triglycerides (TGs), total cholesterol (TC), low-density lipoprotein cholesterol (LDLc), very-low-density lipoprotein cholesterol (VLDLc), and normal or reduced high-density lipoprotein cholesterol (HDLc) (Castelló, 2002; Del Castillo et al., 2004; Mikolasevic et al., 2017). Dyslipidemia in renal transplantation partly results from the use of immunosuppressive medication that interferes with LDLc binding to low-density lipoprotein receptor (LDLR) and corticosteroids that increase cholesterol biosynthesis (Jurewicz, 2003; Agarwal and Prasad, 2016; Shrestha et al., 2019). In addition, hepatic shedding of the lipoprotein clearance receptor syndecan-1 has been associated with increased serum TG levels after renal transplantation (Adepu et al., 2014). Similarly, increased proprotein convertase subtilisin/kexin type-9 (PCSK9)-mediated LDLR degradation is known to increase cholesterol levels in chronic kidney diseases and renal transplant recipients (Eisenga et al., 2017; Pavlakou et al., 2017). Novel data on PCSK9 revealed that hepatic heparan sulfate proteoglycans act as liver-specific co-receptors for PCSK9 and orchestrate PCSK9-mediated LDLR degradation (Gustafsen et al., 2017). We recently showed increased PCSK9–heparan sulfate interaction in various proteinuric renal rat models (Shrestha et al., 2021a; Shrestha et al., 2021b). These findings showed that heparins and heparinoids can interact with PCSK9 *via* their negatively charged sulfated sugar groups and therefore bear the potential to be developed as PCSK9 inhibitors for the treatment of dyslipidemia (Gustafsen et al., 2017; Shrestha et al., 2021a; Shrestha et al., 2021b).

Interestingly, treatment with heparin(oids) is found to improve serum lipid levels (TG and TC) in patients on renal replacement therapy (Elisaf et al., 1997; Lazrak et al., 2017). The effects of heparin(oids) in reducing plasma TG levels are attributed to release/activation of lipoprotein lipase and hepatic lipase (Katopodis et al., 2007); however, the mechanism(s) underlying TC reduction is not clear yet. In addition, both the non-anticoagulant RO-heparin and N-acetylated heparin (NAc-heparin) derivatives are found to inhibit syndecan-1 shedding, another potential mechanism behind lipid reduction (Levidiotis et al., 2004; Ritchie et al., 2011). On the contrary, a number of studies show an increase in TG and TC, and a decrease in serum lipolytic activity, resulting in the accumulation of chylomicrons and lipoproteins on treatment with unfractionated heparin (Akiba et al., 1992; Weintraub et al., 1994).

Therefore, in this study, we investigated 1) the effect of heparin and non-anticoagulant heparinoids on plasma values of lipids, syndecan-1, and PCSK9; 2) the efficacy of heparin and non-anticoagulant heparinoids to prevent the development of glomerulosclerosis and arterial neo-intima; and 3) the association of plasma lipid levels with plasma syndecan-1, PCSK9, and degree of glomerulosclerosis and arterial neo-intima formation in a rat CTD model.

## Materials and Methods

### Animals

In this study, 38 10-week-old female inbred Dark *Agouti* (DA) rats (donors) and 38 10-week-old male inbred Wistar Furth (WF) rats (recipients) were used. DA and WF rats were obtained from Harlan Nederland (Zeist, Netherlands) and Charles River Laboratories (I’Arbresle, Cedex, France), respectively. The local animal Ethics Committee of the University of Groningen approved all the procedures used in the study, and the principles of Laboratory Animal Care (National Institute of Health publication no. 86-23) were followed.

### Kidney Transplantation

Kidney allotransplantation was performed from female DA donors to male WF recipients according to standard procedures as described previously (Rienstra et al., 2009) and immunological data that have been published before (Talsma et al., 2017).

### Treatments and Experimental Groups

In this study, interventions were performed by daily (s.c.) injections of 2 mg/kg BW/day with regular unfractionated heparin (heparin; MW 21,116 Da; *n* = 9) and two non-anticoagulant heparinoids derived from regular heparin: N-desulfated, N-reacetylated heparin (NAc-heparin; MW 18,269 Da; *n* = 10) and periodate-oxidized, borohydride-reduced heparin (RO-heparin; MW 16,522 Da; *n* = 9) as reported before by Talsma et al. (2017) (Casu et al., 2004; Naggi et al., 2005). Although MWs are not exactly identical, these values stay far away from the molecular sizes of low molecular weight (LMW) heparins, which range from 4,500 to 6,000 Da (Akhtar et al., 2018). The treatment dose was chosen according to previous studies and is in the physiological range normally used for the treatment of thrombotic complications as reported previously (Kusyk et al., 2005; Gottmann et al., 2007; Talsma et al., 2017). Production and characterization of these non-anticoagulant heparin derivatives have been described before (Casu et al., 2002; Casu et al., 2004). NAc-hep and RO-heparin were selected because both of these non-anticoagulant heparinoids exert similar anti-inflammatory properties as heparin (Gao et al., 2005; Chen et al., 2009). In addition, biostability, activity, and specificity of these heparinoids are properly controlled (Gao et al., 2005; Chen et al., 2009), and these preparations have been reported to be beneficial in several experimental models as well (Braun et al., 2001; Nakamura et al., 2001; Gottmann et al., 2007). Furthermore, these heparinoid preparations can be produced in larger quantities within affordable costs and limited time. NAc-heparin and RO-heparin differ mainly in their sulfation degree. In RO-heparin, glycol splitting occurred only at the level of pre-existing non-sulfated GlcA and IdoA residues, which corresponds to about 25% of total uronic acids of the parent heparin, yielding non-anticoagulant heparinoid with the same degree of sulfation as regular heparin (Casu et al., 2004). Whereas in NAc-heparin, ∼100% of N-SO3 groups are replaced by N-acetyl groups, drastically reducing their sulfation degree (Naggi et al., 2005). These preparations have been described extensively by the Ronzoni group (Casu et al., 2002; Casu et al., 2004; Naggi et al., 2005; Guerrini and Bisio, 2012). The control transplanted group (*n* = 10) received daily saline injections.

### Clinical Variables

Animals were weighed every day and observed for signs of decreasing animal welfare reflecting their clinical condition as reported by Talsma et al. (2017). Blood pressure was measured non-invasively with the tail cuff method (CODA; Kent Scientific, Torrington, CT, United States). Rats were placed individually in metabolic cages to obtain 24 h urines. Food and water intake during this period were measured. Non-fasting blood sampling by orbital puncture was executed before transplantation (baseline), and at 4 and 8–10 weeks (T1 and T2, respectively) after transplantation. Measurements taken between week 8 and week 10 were depicted as 8 weeks after the Tx group (T2). The total follow-up was 65±4 days.

### Urine and Plasma Analysis

Because of limited amounts of plasma, not all parameters could be measured in all rats. Therefore, in the graphs, we expressed all individual datapoints to make this transparent. Creatinine in plasma and urine and total protein in urine were determined as previously mentioned by Yazdani et al. (2015). Plasma PCSK9 and syndecan-1 levels were determined using the Rat PCSK9 ELISA Kit (CSB-EL017647RA, Cusabio biotech, Houston, TX, United States) and Rat syndecan-1 ELISA kit (CSB-E17115r, Cusabio biotech, Houston, TX, United States) as per manufacturer’s instructions.

### Lipid Measurements

TC and TG levels were measured with a colorimetric assay as reported before by Wijers et al. (2019). In brief, TC levels were determined using colorimetric assays (11489232, Roche) with cholesterol standard FS (DiaSys Diagnostic Systems GmbH) as a reference. TG levels were measured using a Trig/GB Kit (1187771, Roche) with Roche Precimat Glycerol standard (16658800) as a reference.

### Fast-Protein Liquid Chromatography

Rat plasma samples were fractionated by fast protein liquid chromatography (FPLC) as previously described (Gerdes et al., 1992) with minor modifications in order to investigate the changes in lipoprotein particles (VLDL, LDL, and HDL). In brief, the system contained a PU-980 ternary pump with an LG-980-02 linear degasser and a UV-975 UV/VIS detector (Jasco). EDTA plasma was diluted 1:1 with tris-buffered saline, and 300 μl sample/buffer mixture was loaded onto a Superose 6 HR 10/300 column (GE Healthcare, Life Sciences Division) for lipoprotein separation at a flow rate of 0.5 ml/min. Quantitative analysis of the chromatograms was performed with ChromNav chromatographic software, version 1.0 (Jasco). The plots for individual fast-performance liquid chromatography profiles were generated using GraphPad version 8.0.1.

### Quantification of Glomerulosclerosis and Neo-Intima Formation

Identification and quantification of glomerulosclerosis and neo-intima formation in kidneys were determined with periodic acid-Schiff (PAS) and Verhoeff’s staining, respectively, and blindly scored as mentioned previously by Rienstra et al. (2010).

The sections were semi-quantitatively scored for focal glomerulosclerosis in a blinded fashion by determining the level of mesangial expansion and focal adhesion in each quadrant in a glomerulus and expressed on a scale from 0 to 4. If the glomerulus was unaffected, it was scored as 0; if one quadrant of the glomerulus was affected, it was scored as 1, two affected quadrants as 2, three affected quadrants as 3, and 4 affected quadrants as 4. In total, 50 glomeruli per kidney were analyzed, and the total FGS score was calculated by multiplying the score by the percentage of glomeruli with the same FGS score. The sum of these scores gives the total FGS score with a maximum of 400.

Neo-intima formation was scored at 200× magnification (Olympus B×50; Olympus Europa, Hamburg, Germany) by determining the percentage of luminal occlusion. All the identifiable elastin positive intrarenal vessels were evaluated in a blinded fashion. The lumen of vessels was the mean length of two straight lines drawn from the internal elastic lamina (IEL) and passing through the center of the vessel. The areas enclosed by the lumen, internal elastic lamina, and external lamina were measured. The area between the lumen and internal elastic lamina was described as the neo-intimal area. The percentage of neo-intimal area to the area enclosed by IEL (total lumen) is described as the percentage of luminal occlusion.

### Statistics

Analyses were performed using GraphPad version 8.0.1 (GraphPad software). The one-way ANOVA test and the Kruskal Wallis test were used to compare differences between the groups. When significant differences were observed between the means, Dunnett’s multiple comparison test, corrected for multiple comparisons, or Dunn’s multiple comparison test, corrected for multiple comparisons, was used as post-test to identify which specific means were significant from the others (based on normality of data). Data are given as means ± SEM. The non-parametric Spearman correlation was used to analyze the association between parameters. For all experiments, a *p* value of <0.05 was considered statistically significant.

## Results

### Development of CTD-Related Renal Failure

This study included 38 male WF rats that were transplanted with a female DA kidney as reported before (Naggi et al., 2005). Renal graft loss was evidenced by clinical signs such as pilo-erected fur, severe body weight loss, disoriented behavior, and high blood creatinine values. Six rats that had to be sacrificed before the end of the experiment (before 8–10 weeks after transplantation) were excluded from all histological and biochemical analyses described later. Graft loss among the various heparinoid groups was not significantly different. Accordingly, the following groups with mentioned group size were studied: allografts treated with saline (*n* = 8), heparin (*n* = 8), N-acetyl heparin (*n* = 8), and RO-heparin (*n* = 8). In the plasmas of the rats taken at 8 weeks after renal transplantation, 4 h after heparin(oid) injection, we measured the activated partial thromboplastin time. In the saline-treated transplanted rats, this was 75 s (median value). In the regular heparin group, this time was 173 s (saline versus regular heparin: *p* < 0.05), 73 s in the RO-heparin group, and 69 s in the N-acetyl heparin group (both non-anticoagulant heparinoids not being different from saline-treated rats) (not shown). These data show that both chemically modified heparin preparations indeed were non-anticoagulant, resulting in a similar activated partial thromboplastin time compared with saline-treated rats.

### Effects of (Non-)Anticoagulant Heparin(oids) on Clinical Parameters

#### Body Weight, Mean Arterial Blood Pressure, Food and Water Intake, and Urinary Output

Treatment with heparin and non-anticoagulant heparins had no effect on body weight (Supplementary Table S1), food and water intake, and urine output (not shown) of the WF recipient rats at 8 weeks (Supplementary Table S1). The mean arterial blood pressure increased gradually in recipient WF rats until the end of the experiment without statistical significances among the groups (Supplementary Table S1).

#### Renal Function

Saline-treated groups developed CTD-related renal failure as evidenced by reduced creatinine clearance and a rise in both serum creatinine and urinary protein excretion (Supplementary Table S1). Serum creatinine levels increased and creatinine clearance significantly reduced in all groups over time, without differences among the groups. No differences were observed in proteinuria between the groups after 8 weeks follow-up (Supplementary Table S1), indicating that treatment with heparin and non-anticoagulant heparinoids had no effect on renal function.

### Effects of (Non-)Anticoagulant Heparin(oids) on Lipid Levels

#### Serum Triglycerides (TG) Levels

**FIGURE 1:**
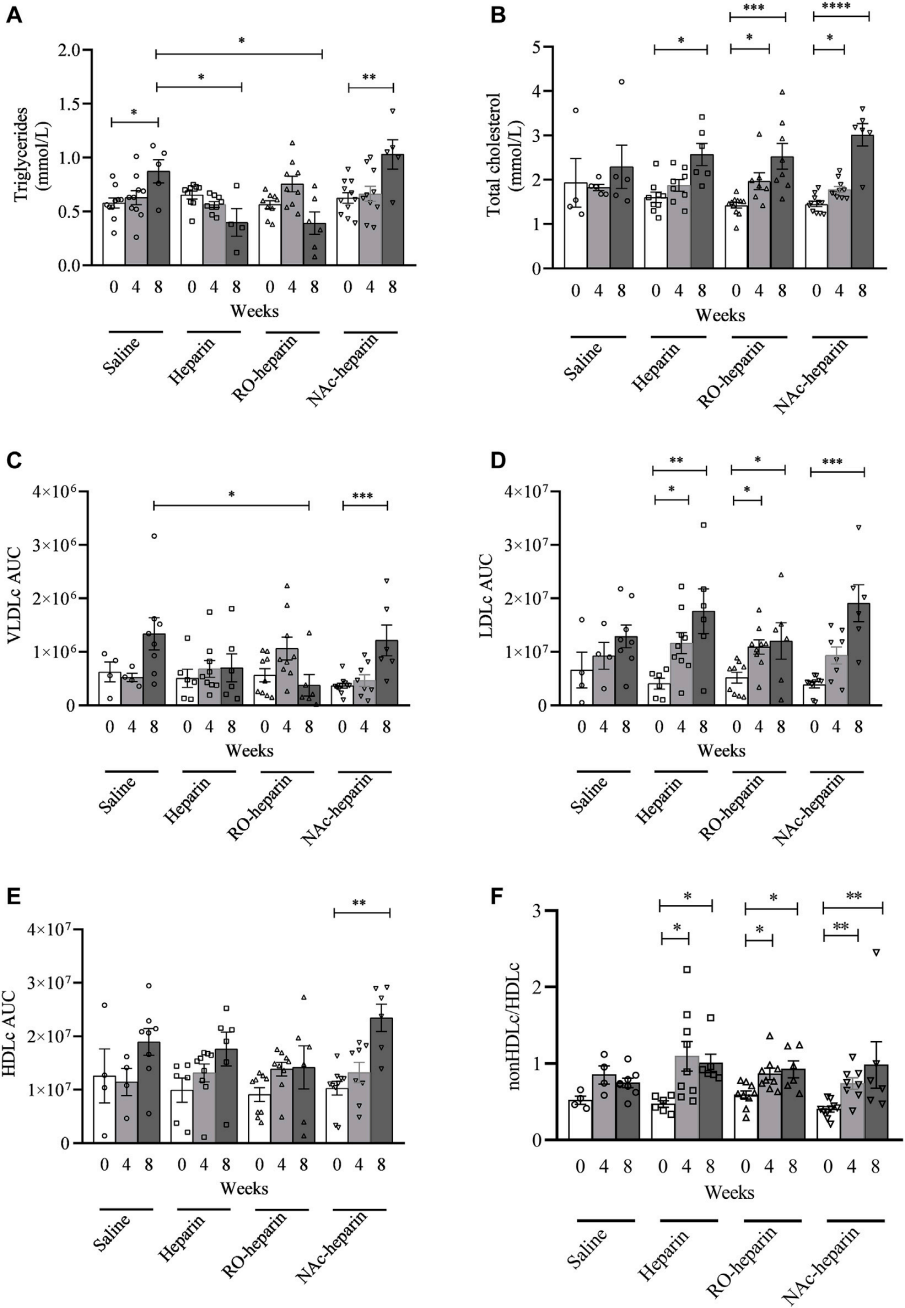
Lipid measurements in saline-treated, heparin-treated, RO-heparin-treated, and NAc-heparin-treated groups at baseline, 4 weeks after Tx, and 8 weeks after Tx. **(A)** Serum triglycerides. **(B)** Total cholesterol. **(C)** VLDLc AUC (area under the curve). **(D)** LDLc AUC. **(E)** HDLc AUC. **(F)** non-HDLc/HDLc. *p < 0.05. Data are shown as mean ± SEM.

To investigate the effects of heparin and non-anticoagulant heparinoids on serum lipid levels, serum TG and TC levels were measured. Serum TG levels were significantly increased in saline- and NAc-heparin-treated groups over time, whereas heparin and RO-heparin treatment prevented the increase in serum TG levels over time (Figure 1A). At 8 weeks, serum TG levels in heparin- and RO-heparin-treated groups were significantly lower than those in saline-treated groups (both *p* < 0.05; Figure 1A).

#### Serum Cholesterol Levels

No significant differences in serum TC levels were observed in saline-treated group over time, although values tend to increase over time (Figure 1B). All treated groups (heparin, RO-heparin, and NAc-heparin groups) showed a significant increase in serum TC over time (Figure 1B). However, at 8 weeks, TC values were not significantly changed in any of the treatment groups compared to the saline-treated group (Figure 1B), indicating no effects of heparin, RO-heparin, and NAc-heparin treatments on serum TC levels in the CTD rat model.

**FIGURE 2:**
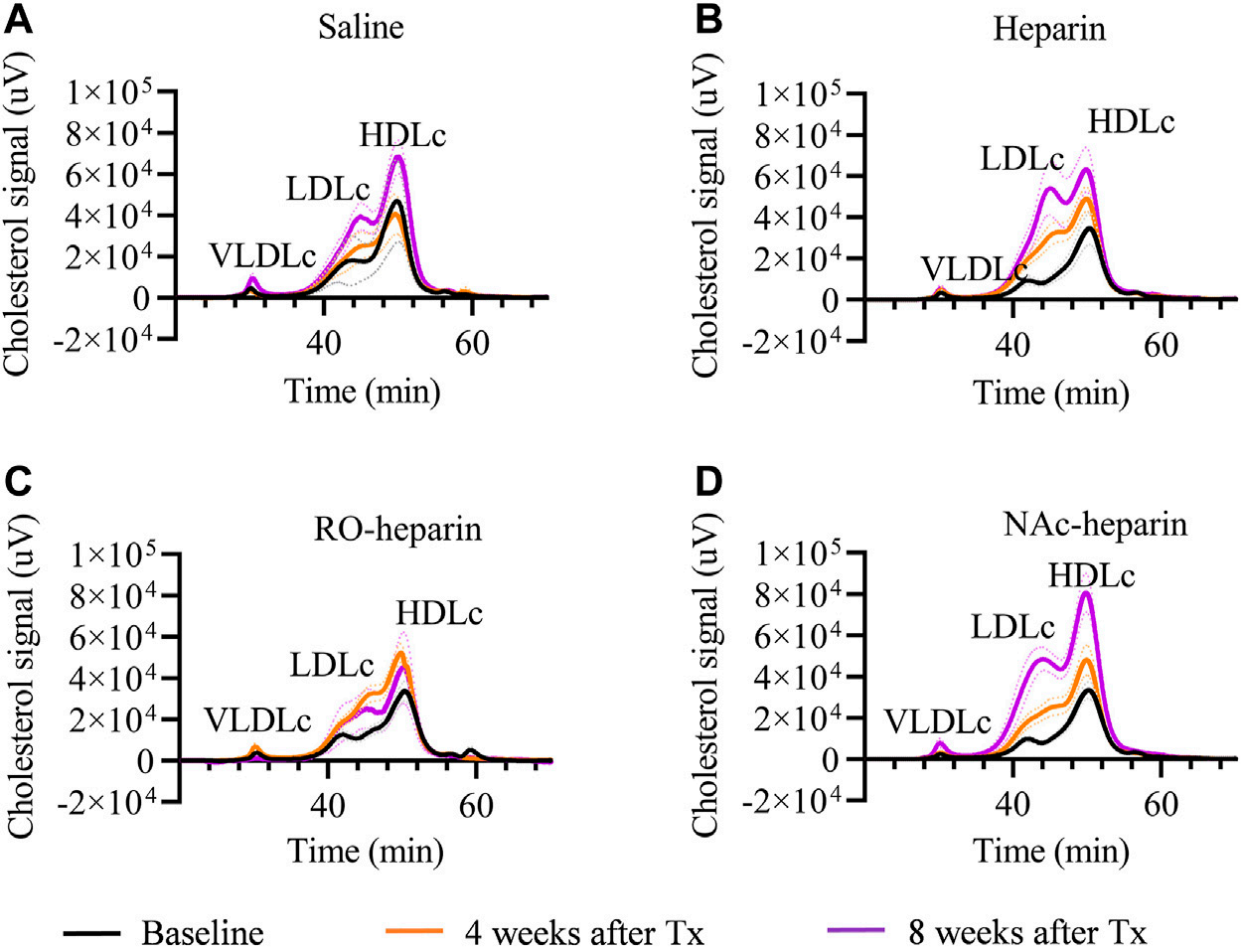
Cholesterol profiling by FPLC in saline-treated, heparin-treated, RO-heparin-treated, and NAc-heparin-treated group at baseline, 4 weeks after Tx, and 8 weeks after Tx. **(A)** Saline-treated group, **(B)** heparin-treated group, **(C)** RO-heparin-treated group, and **(D)** NAc-heparin-treated group. The dark lines indicate the mean, and the dotted lines indicate SEM.

To get more insight into cholesterol profiles, we further profiled serum lipoproteins levels (Figures 2A–D). VLDLc levels followed the same pattern as TG levels (ANOVA: *p* < 0.05). VLDLc levels were increased in saline and NAc-heparin (*p* < 0.01) treated groups, whereas heparin and RO-heparin treatment prevented the increase in VLDLc levels over time (Figure 1C). At 8 weeks, RO-heparin-treated groups showed a significant reduction in serum VLDLc levels compared to the saline-treated group (*p* < 0.05; Figure 1C). Then the same tendency (although not statistically significant) was seen in the heparin group. As expected for TG-rich VLDL lipoproteins, serum VLDLc levels were positively correlated with serum TG levels at 8 weeks (*r* = 0.6, *p* = 0.005) (not shown).

LDLc and HDLc levels were increased in all groups over time (Figures 1D,E). At 8 weeks, no significant differences were observed in LDLc and HDLc levels in all treatment groups as compared to the saline-treated group. Likewise, the non-HDLc/HDLc ratio was increased in all groups (Figure 1F). No significant differences were observed in the non-HDLc/HDLc ratio in any of the treatment groups compared to the saline-treated group at 8 weeks (Figure 1F).

Altogether, these data indicate that heparin and RO-heparin treatment prevented an increase in serum TG and VLDLc levels without affecting the LDLc, HDLc, and non-HDlc/HDLc ratio in the CTD model.

### Effects of (Non-)Anticoagulant Heparin(oids) on Serum PCSK9 Levels

**FIGURE 3:**
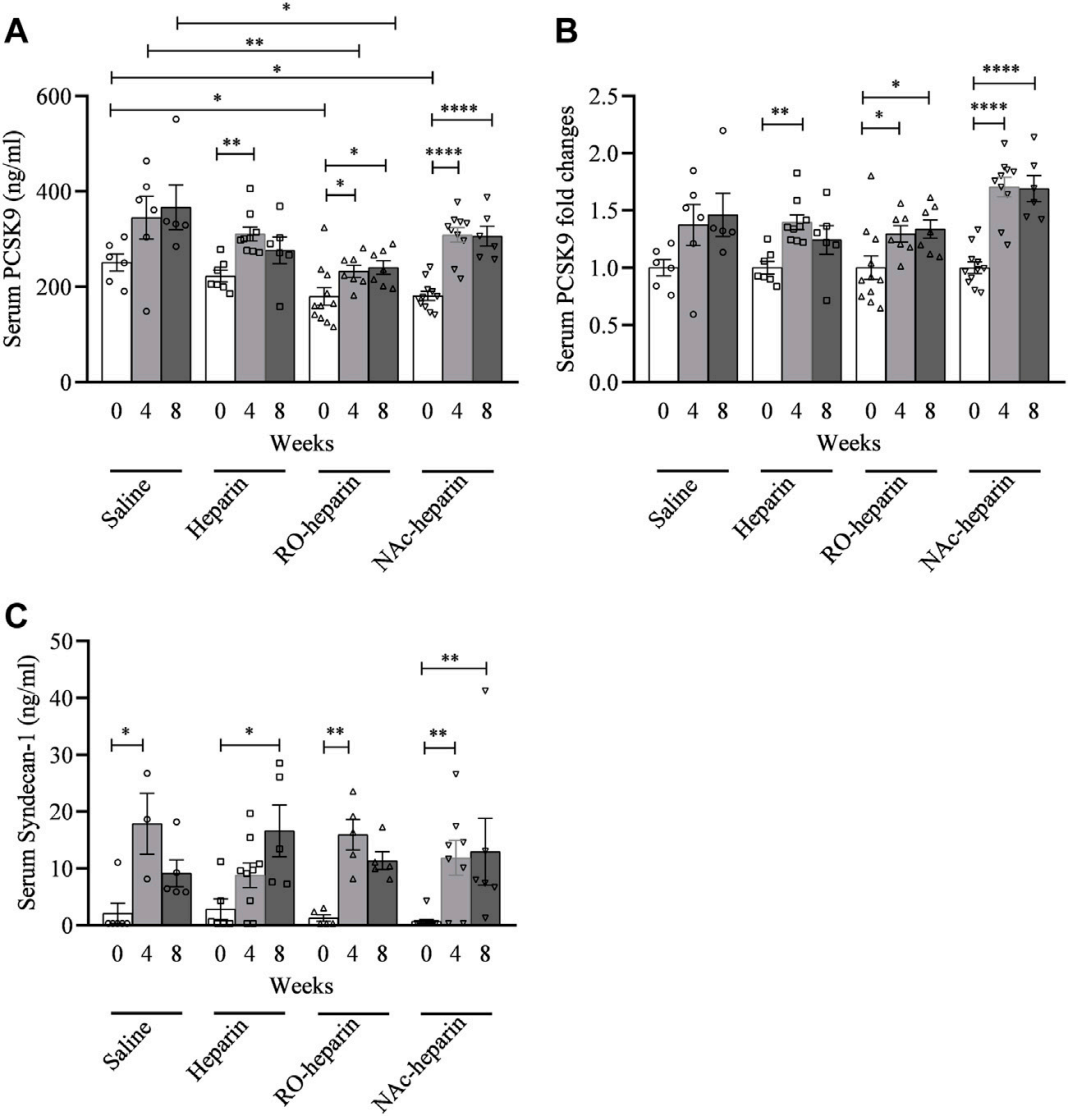
Serum PCSK9 and serum syndecan-1 levels in saline-treated, heparin-treated, RO-heparin-treated, and NAc-heparin-treated groups. **(A)** Serum PCSK9 levels, **(B)** serum PCSK9 expressed as fold changes, and **(C)** serum syndecan-1 levels. Data are shown as mean ± SEM.

Serum PCSK9 levels in the RO-heparin treated group and the NAc-heparin treated group were significantly lower than those in the saline-treated group at baseline (Figure 3A). Because of variation in baseline PCSK9 levels in the various groups, serum PCSK9 levels were adjusted for their respective baseline values and expressed as fold change. We observed ∼1.5-fold increments in serum PCSK9 levels in all groups over time (Figure 3B). When we compared serum PCSK9 levels in heparin-, RO-heparin-, and NAc-heparin-treated groups with saline-treated groups at 8 weeks, no significant differences were observed (Figure 3B), indicating no effects of heparin and heparinoids on serum PCSK9 levels. Similarly, no association of serum PCSK9 levels was observed with serum lipid levels (TG, TC, VLDLc, LDLc, HDLc, and non-HDLc/HDLc) and with renal function parameters (creatinine clearance, proteinuria, and serum creatinine) at 8 weeks (not shown), indicating that serum PCSK9 levels might not be a crucial determinant for dyslipidemia in our rat model of CTD.

### Effects of (Non-)Anticoagulant Heparin(oids) on Serum Syndecan-1 Levels

We previously reported syndecan-1 shedding in the same transplant model as used here (Adepu et al., 2014). Serum syndecan-1 levels were found to be increased ∼10-fold in all groups over time (Figure 3C). At 8 weeks, heparin-, RO-heparin-, and NAc-heparin-treated groups showed no significant differences in serum syndecan-1, compared to the saline-treated group (Figure 3C). Furthermore, serum syndecan-1 levels at 8 weeks were positively associated with TC, LDLc, and HDLc levels (*r* = 0.5, *p* = 0.03; r = 0.49, *p* = 0.03; and *r* = 0.49, *p* = 0.03, respectively) but not with TG, VLDLc, and renal function parameters (not shown).

### Development of Glomerulosclerosis and Transplant Arteriopathy

**FIGURE 4:**
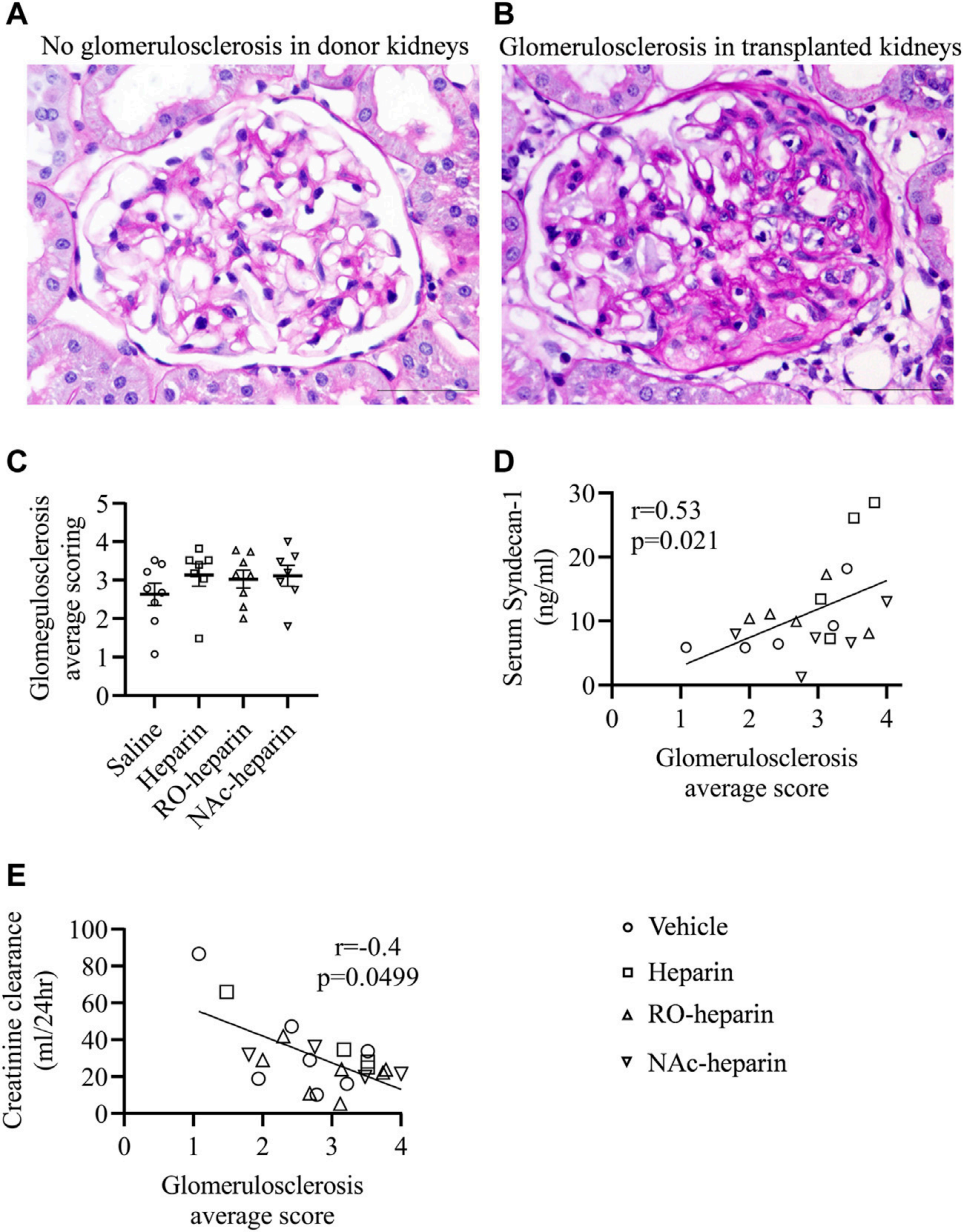
Glomerulosclerosis in saline-treated, heparin-treated, RO-heparin-treated, and NAc-heparin-treated groups. **(A,B)** Representative pictures of glomerulosclerosis in non-transplant donor kidney and transplanted kidney at 8 weeks. **(C)** Glomerulosclerosis scoring on kidneys of saline-treated, heparin-treated, RO-heparin-treated, and NAc-heparin-treated groups at 8 weeks. **(D)** Univariate correlation analysis of glomerulosclerosis with serum syndecan-1. **(E)** Univariate correlation analysis of glomerulosclerosis with creatinine clearance. Data are shown as mean ± SEM. Scales represent 50 μm.

Two of the major histopathological signs of CTD are glomerulosclerosis and transplant arteriopathy (Najafian and Kasiske, 2008). Therefore, we examined glomeruli from saline-, heparin-, and heparinoid-treated groups at 8 weeks and scored them for the presence of glomerulosclerosis. At 8 weeks, all groups showed glomerulosclerosis with variability among animals; however, no differences were observed (Figures 4A–C). Non-transplanted rats had a glomerulosclerosis score close to zero (Figure 4A), indicating that heparin and non-anticoagulant heparinoids are not effective in reducing glomerulosclerosis in this fully HLA-mismatched model with severe renal damage. However, glomerulosclerosis scores were significantly positively associated with serum syndecan-1 levels (*r* = 0.53, *p* = 0.021) (Figure 4D) and negatively associated with creatinine clearance (*r* = −0.4, *p* = 0.049, respectively) (Figure 4E).

**FIGURE 5:**
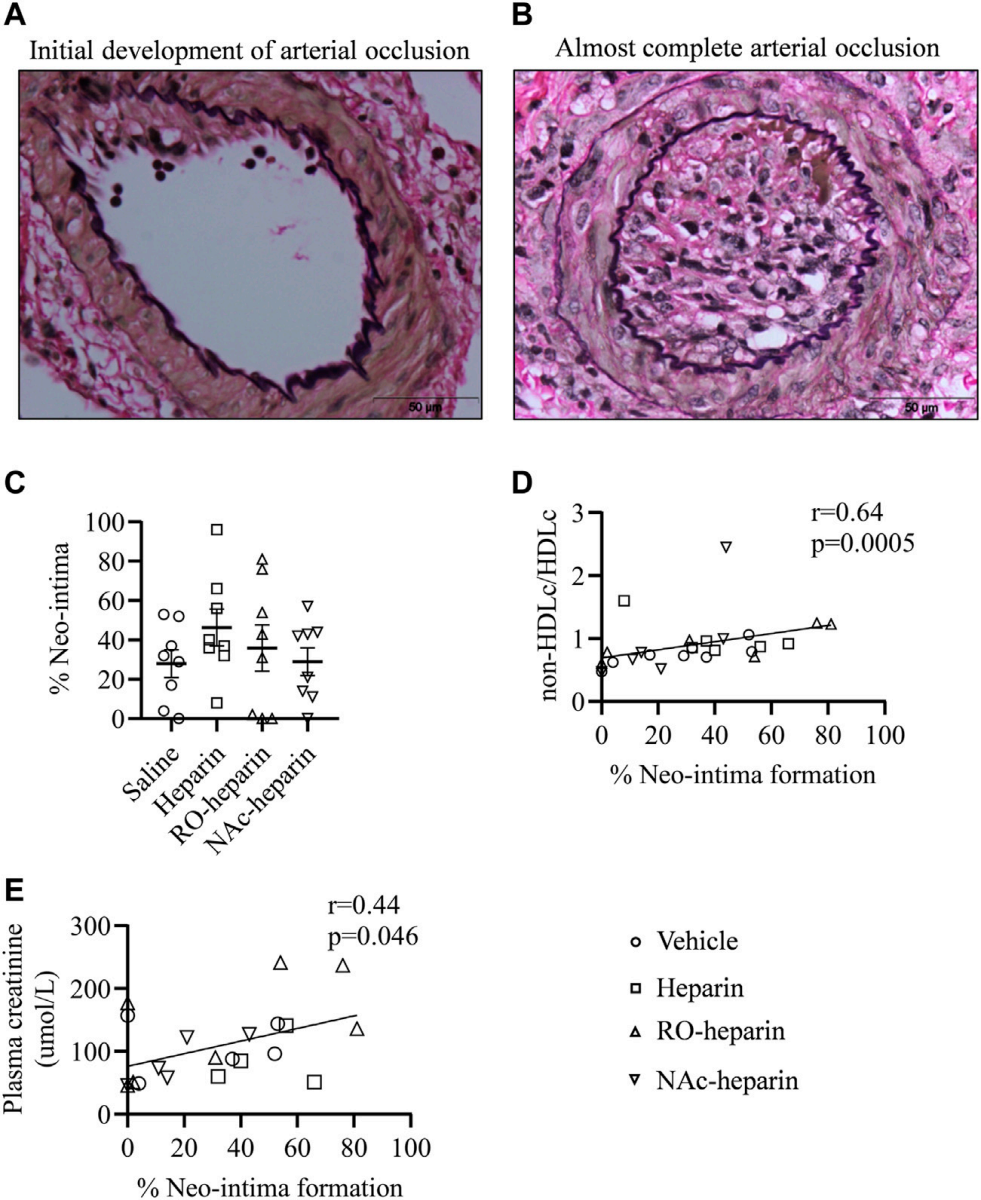
Transplant arteriopathy in saline-treated, heparin-treated, RO-heparin-treated, and NAc-heparin-treated groups. **(A,B)** Representative pictures for variability in neo-intima formations. **(C)** Percentage of neo-intima formation in the arteries of saline-treated, heparin-treated, RO-heparin-treated, and NAc-heparin-treated groups at 8 weeks. **(D)** Univariate correlation analysis of percentage of neo-intima with non-HDLc/HDLc. **(E)** Univariate correlation analysis of percentage of neo-intima with serum plasma creatinine. Data are shown as mean ± SEM. Scales represent 50 μm.

Next, we evaluated the renal arteries of allografts from saline-, heparin-, and heparinoid-treated groups at 8 weeks for arterial neo-intima formation and analyzed if the reduction in TG and VLDLc levels by heparin and RO-heparin lowered the risk of arterial lumen occlusion (neo-intima formation). All treated groups showed variable lumen occlusion due to neo-intima formation. No significant differences were observed in neo-intima formation in any of the treatment groups compared to the saline-treated group (Figures 5A–C). Interestingly, the percentage of neo-intima formation was significantly positively associated with non-HDLc/HDLc levels (*r* = 0.64, *p* < 0.001) (Figure 5D), suggesting a role of bad cholesterol profile in neo-intima formation over TG levels. Similarly, significant positive associations were observed with plasma creatinine (*r* = 0.44, *p* = 0.046) (Figure 5E).

## Discussion

In a rat model of CTD, we show that heparin and non-anticoagulant heparinoid (RO-heparin) could prevent the increase in serum TG and the TG-rich VLDL particles. None of the heparin(oids), however, could prevent a progressive increase in TC, LDLc, HDLc, serum PCSK9, and serum syndecan-1 levels. Similarly, transplant glomerulosclerosis and arterial neo-intima formation could not be attenuated by heparin(oid) treatment. Arterial neo-intimal scores were positively associated with unfavorable cholesterol profile (non-HDLc/HDLc ratio). Transplant glomerulosclerosis was positively associated with shedding of the lipoprotein clearance receptor syndecan-1. These data suggest that development and progression of CTD are independent of changes in TGs and VLDLc levels, rather related to syndecan-1 shedding and cholesterol profile.

Although we did not study the lipoprotein lipase (LPL) activity in our model, the effects of heparin in reducing plasma TG and VLDLc levels might be attributed to the release/activation of lipoprotein lipase and hepatic lipase as reported before (Davenport, 2009).

We observed >10-fold increase in serum syndecan-1 levels in saline as well as heparin/non-anticoagulant heparin-treated groups over time. Furthermore, we observed no effects of heparin and non-anticoagulant heparinoids on serum syndecan-1 levels despite strong reductions in TG and VLDLc levels. It is worthwhile to mention that serum syndecan-1 levels at 8 weeks are strongly positively associated with TC, LDLc, and HDLc levels, but also with glomerulosclerosis. These data indicate that increased syndecan-1 shedding leading to reduced lipoprotein clearance might be a cause underlying dyslipidemia and glomerulosclerosis in our model. Although reported by others (Ritchie et al., 2011), neither of the heparins were effective to prevent syndecan-1 shedding in our model, indicating TG and VLDLc lowering effects by heparin/RO-heparin do not involve syndecan-1. In addition, previously, we showed increased plasma values of syndecan-1 associated with reduced kidney function (plasma creatinine, creatinine clearance, and degree of proteinuria) (Adepu et al., 2014; Adepu et al., 2015). In general, loss of renal function is associated with increased fibrosis. So, these data in human renal transplant recipients are in line with our current positive association of plasma syndecan-1 with glomerulosclerosis.

Tissue remodeling and invasion of the neo-intima into the vascular lumen due to inflammatory processes inside the walls of arteries are important hallmarks of CTD. The interaction of lipids such as oxidized LDL and immune cells is thought to be a driving force behind chronic inflammation of the arterial wall during atherogenesis. Accumulation of lipid particles in vessel walls and subsequent immunological response cause plaque formation, which gets aggravated in dyslipidemic situations (Schaftenaar et al., 2016; Sandesara et al., 2019). Elevated serum cholesterol levels, particularly LDLc levels, have been implicated in the worsening of vascular lesions and neo-intima formation in experimental models of CTD and in a randomized clinical trial (Silverman et al., 2016). Hypercholesterolemia post-renal transplantation has been associated with progressive deterioration of graft function, higher proteinuria, and aggravated histological lesions in allografts (Wissing et al., 2000), making hypercholesterolemia an independent risk factor for kidney allograft loss (Roodnat et al., 2000; Öörni et al., 2019). Recently, TG-rich lipoproteins such as chylomicrons, VLDL, and their remnants are found to increase endothelial inflammation and promote atherogenesis independently of LDLc (Quaschning et al., 1999; Generoso et al., 2019). Furthermore, immunosuppressives used in renal transplant patients cause accumulation of TG-enriched VLDL and LDL particles (Quaschning et al., 1999). However, despite the effective reduction of TG and VLDLc by heparin and RO-heparin, we failed to observe beneficial effects on CTD. This could be due to a strong inflammatory and allo-immune response to HLA mismatch in our CTD rat model, with lipid levels playing a minor role. As a result, the beneficial effects of TG and VLDLc reduction could not be seen as clearly. In addition, dyslipidemia itself might be caused due to strong systemic inflammation in our rat model as previously observed in several inflammatory diseases (Esteve et al., 2005). Therefore, although lipids undoubtedly contribute to the development and progression of atherosclerosis, the strong inflammatory response in our rat transplantation model mainly orchestrates the progression and outcome of the disease.

Apart from the role of dyslipidemia in transplant vasculopathy, dyslipidemia also induces glomerulosclerosis. Various studies have shown the accumulation of lipoproteins in glomerular mesangium, which accelerates matrix production in several rat models of renal diseases (Kasiske et al., 1991; Wang et al., 2005). Moreover, TG-enriched oxidized LDLc also stimulates the synthesis of cytokines, growth factors, and extracellular matrix proteins in glomerular mesangial cells, eventually leading to glomerulosclerosis (Fellström, 1999), indicating both TG and TC to participate in the development and progression of CTD. On the contrary, there are other studies that have failed to establish any relationship among elevated lipid levels, functional deterioration of kidney grafts, and CTD (Massy et al., 1996; Hillebrand et al., 1999), making the role of dyslipidemia in CTD a matter of debate. Our study shows that despite the effective reduction of TGs and VLDLc levels by the treatment with heparin and RO-heparin, no beneficial effects were observed on renal function, glomerulosclerosis, and neo-intima formation. Furthermore, the effects of heparin and heparinoids in reducing lipid levels have been controversial. Previous studies in patients have shown variable effects of heparin and low molecular weight heparins on serum lipid levels, which range from reduction to aggravation of lipid levels (Katopodis et al., 2007), likely dependent on patient-related variables. In our renal transplantation model, neo-intima formation was significantly positively associated with non-HDLc/HDLc levels, suggesting a contribution of a bad cholesterol profile to neo-intima formation, however, without any improvement by heparin(oid) treatment. We realize that our fully HLA mismatched rat CTD model shows a very strong allo-immune response. Several studies in rats have reported that treatment of allograft recipients with immunosuppressants like cyclosporin A and rapamycin can block the development of CTD-related vasculopathy (Orosz and Pelletier, 1997). Similarly, proteoglycans such as HSPGs have long been known to have a role in the inflammatory process, owing to the binding of HSPGs to L-selectins and P-selectins found on leukocytes and endothelial cells, as well as the presentation and gradient generation of chemokines (Ivetic et al., 2019). As a result, therapeutic interventions based on the proteoglycan chemokine interaction have been developed. Treatment with some low molecular weight heparins has been demonstrated to minimize the signs of increasing renal failure in experimental renal transplantation (Persson, 1988; Braun et al., 2001; Rienstra et al., 2010). The authors found that LMW heparin lowers renal monocyte, T-cell, and major histocompatibility complex II positive (MHCII+) infiltration in these experiments. Our previous study by Talsma et al. (2017) showed no positive effect of heparin/non-anticoagulant heparin derivative treatment on graft function and outcome. Furthermore, RO-heparin treatment in transplantation indeed reduced renal leukocyte recruitment, but increased the migration of antigen-presenting cells to newly formed lymphatic vessels in transplanted kidneys (Talsma et al., 2017). The differences in research outcomes could be explained by the fact that we used unfractionated heparin/non-anticoagulant heparin derivatives and used a full HLA mismatch model, whereas Braun et al. (2001) used LMW heparins, a milder Lewis to Fisher model. Therefore, it seems that CTD in our renal transplant model is primarily driven by immunological responses. The lack of clinically significant effects on CTD by controlling TG and VLDL levels using heparin therefore might be a strong immune component in the current study. Therefore, future investigations are needed in proteinuric rat models where immune responses are subtle and might help to understand the effects of heparin(oids) on serum lipids and PCSK9 levels (Shrestha et al., 2021a; Shrestha et al., 2021b). Future research could also investigate differential responses of unfractionated *versus* LMW heparin/non-anticoagulant heparin derivatives.

In conclusion, the efficacy of heparin and non-anticoagulant heparins in lipid reduction is heterogeneous, controversial, and context dependent. Furthermore, complete relying on heparin and non-anticoagulant heparins in preventing CTD and CTD-related tissue remodeling might not be warranted by our results, at least not in the transplantation setting. Future investigations on non-immunological proteinuric animal models and human nephrotic diseases might be superior to target PCSK9/syndecan-1/cholesterol axis.

## Data Availability

The raw data supporting the conclusion of this article will be made available by the authors, without undue reservation.

## Abbreviations

BW, body weight; CTD, chronic transplant dysfunction; HSPG, heparan sulfate proteoglycans; MW, molecular weight; PCSK9, proprotein convertase subtilisin/kexin type-9; LDLR, low-density lipoprotein receptor; LRP-1, low-density lipoprotein receptor related protein 1; TC, total cholesterol; TGs, triglycerides; LDLc, low-density lipoprotein cholesterol; VLDLc, very-low-density lipoprotein cholesterol; HDLc, high-density lipoprotein cholesterol.
