# Supplementary material for: Prevention of Triglyceridemia by (Non-)Anticoagulant Heparin(oids) Does Not Preclude Transplant Vasculopathy and Glomerulosclerosis
Source: Front Cell Dev Biol. 2022 Mar 7;10:798088. doi: 10.3389/fcell.2022.798088 (PMC8957085; doi:10.3389/fcell.2022.798088)
Supplement: Supplementary file 1 [file Table1.DOCX]

**Supplementary Table 1: Clinical characteristics of saline treated, heparin, RO-heparin and N-acetylated heparin treated rats with CTD.**

|  |  | DA-to-WF allograft | | | |
| --- | --- | --- | --- | --- | --- |
|  |  | Saline (n=8) | Heparin (n=8) | RO-heparin (n=8) | N-acetylated heparin (n=8) |
| Body weight (g)^♣^ | Baseline | 286 ± 2 | 273 ± 7 | 271 ± 4 | 262 ± 1 |
|  | 4 weeks after Tx | 293 ± 8 | 296 ± 7 | 289 ± 6.6 | 282 ± 6 |
|  | 8 weeks after Tx | 323 ± 12^#^ | 310 ± 12 | 305 ± 11.62^#^ | 300 ± 8^##^ |
| Mean blood pressure (mmHg) | Baseline | 115 ± 9 | 115 ± 9 | 115 ± 9 | 115 ± 9 |
|  | 4 weeks after Tx | 114 ± 5 | 123 ± 7 | 134 ± 6 | 137 ± 6 |
|  | 8 weeks after Tx | 146 ± 8 | 148 ± 7^#^ | 146 ± 5^#^ | 161 ± 9^#^ |
| Proteinuria (mg/24hr) | Baseline | 10 ± 1 | 8 ± 1 | 9 ± 1 | 9 ± 1 |
|  | 4 weeks after Tx | 10 ± 2 | 10 ± 2 | 9 ± 1 | 12 ± 5 |
|  | 8 weeks after Tx | 63 ± 17^##^ | 72 ± 10^##^ | 32 ± 6^#^ | 127 ± 6^#^ |
| Plasma creatinine (μmol/L) ^♣^ | Baseline | 20 ± 1 | 19 ± 1 | 16 ± 1 | 17 ± 1 |
|  | 4 weeks after Tx | 101 ± 17^##^ | 84 ± 14^##^ | 146 ± 52^##^ | 119 ± 36^##^ |
|  | 8 weeks after Tx | 107 ± 20^##^ | 84 ± 20^##^ | 140 ± 31^###^ | 85 ± 17^##^ |
| Creatinine clearance (ml/24hr) ^♣^ | Baseline | 69±9 | 63±8 | 63±10 | 57±15 |
|  | 4 weeks after Tx | 32±5^##^ | 39±8 | 24±5^##^ | 15±5^##^ |
|  | 8 weeks after Tx | 35±10^##^ | 38±9 | 22±4^##^ | 30±4 |

Data represent mean and standard error of mean (SEM). At 8 weeks no significant differences were observed between saline treated and heparin(oids) treated groups. # represent comparison between baseline vs 4 weeks and 8 weeks after Tx. # represent *p*<0.05 and ##represent *p* <0.01. There were no significant differences between heparin(oid) treatment groups with saline group. ^♣^Described before in Talsma et al. PLOSONE 2017;12(6):e0180206.
